# Outcomes of Robot-Assisted Transbronchial Biopsies of Pulmonary Nodules: A Review

**DOI:** 10.3390/diagnostics15040450

**Published:** 2025-02-13

**Authors:** Peter A. Ebeling, Salim Daouk, Jean I. Keddissi, Houssein A. Youness

**Affiliations:** 1 Interventional Pulmonary Program, Section of Pulmonary, Critical Care and Sleep Medicine, University of Oklahoma, Oklahoma City, OK 73104, USA; peter-ebeling@ouhsc.edu (P.A.E.); salim-daouk@ouhsc.edu (S.D.); jean-keddissi@ouhsc.edu (J.I.K.); 2 Section of Pulmonary, Critical Care and Sleep Medicine, The Oklahoma City VA Healthcare System, Oklahoma City, OK 73104, USA

**Keywords:** robotic bronchoscopy, diagnostic yield, review, pulmonary nodules

## Abstract

**Background/Objectives**: Robot-assisted bronchoscopy (RAB) is a novel platform for sampling peripheral pulmonary nodules (PPNs). To further clarify the role robot-assisted platforms have in diagnosing PPNs, we performed a review of the recent literature. **Methods**: A systematic review was performed in Medline from 2019 to 2024 using the search terms “robotic bronchoscopy”, “diagnostic yield”, “sensitivity”, and “positive predictive value”, alone and in combination. Studies that focused on earlier electromagnetic bronchoscopies were excluded. The patient demographic information, nodule characteristics, intra-procedure imaging modality, biopsy methods, diagnostic yield, sensitivity for malignancy, and adverse outcomes were analyzed. A total of 22 studies were available for the analyses. **Results**: The diagnostic yield was variable and ranged from 69 to 93%, with a median of 86%. The sensitivity ranged from 69% to 91.7%, with a median of 85%. The effect of the nodule size on the diagnostic yield was variable across the literature. Obtaining an eccentric or concentric view on a radial endobronchial ultrasound (rEBUS) was associated with a higher diagnostic yield than obtaining no view. A nodule appearance on CT imaging and the location were not definitively associated with a higher diagnostic yield. Fine needle aspiration usage ranged from 93.5 to 100%, with a median of 96.95%, while the use of biopsy forceps ranged from 2.7 to 96%, with a median of 69.9%. The most common complication was a pneumothorax, which occurred in 1–5.7% of cases, with a median of 1.6%. **Conclusions**: Robot-assisted transbronchial biopsies produce diagnostic yields that approach those of transthoracic needle aspirations. The nodule location and appearance may not affect the diagnostic yield. Obtaining a concentric or eccentric view on rEBUS is likely associated with an increased diagnostic yield. Additional prospective studies would better inform practitioners as this technology becomes more widespread.

## 1. Introduction

The use of low-dose chest computed tomography (LDCT) to screen for pulmonary malignancies has led to the increased detection of pulmonary nodules in the United States [1]. As the number of detected nodules has increased, so too have referrals for sampling these nodules. Historically, transthoracic needle aspiration (TTNA) has produced higher diagnostic yields, but also higher complication rates compared with bronchoscopic biopsies [2]. The first studies of electromagnetic (EMN) bronchoscopy were published in 2005. EMN bronchoscopy overcame the traditional limitations of flexible bronchoscopy with longer, hyper-flexible catheters and software for navigating to distal airways previously inaccessible to the bronchoscopist. However, the diagnostic yields remained lower than for transthoracic approaches [3,4].

Robot-assisted bronchoscopy platforms have recently gained wider acceptance for sampling peripheral pulmonary nodules (PPNs). The Food and Drug Administration (FDA) granted clearance to Ion^TM^ (Intuitive Surgical, Sunnyvale, CA, USA) in 2019 and Monarch^TM^ (Auris Health, Inc., Redwood City, CA, USA) in 2018. A third platform, the Galaxy System^TM^ (Noah Medical, San Carlos, CA, USA) was granted FDA clearance in 2023. To further clarify the role robot-assisted platforms have in diagnosing PPNs, we performed a review of the recent literature. Here, we present the biopsy outcome and safety data on patients that underwent transbronchial pulmonary nodule biopsies with robot-assisted bronchoscopy.

## 2. Materials and Methods

A systematic review was performed in Medline from 2019 to 2024 using the search terms “robotic bronchoscopy”, “diagnostic yield”, “sensitivity”, and “positive predictive value”, alone and in combination. The references for the included articles were also surveyed for relevant studies.

Both retrospective and prospective studies that contained adult study participants, reported diagnostic yield and sensitivity, and had full text available were included. Studies that focused on earlier electromagnetic bronchoscopies, metanalyses, and review articles were excluded. Patient demographic information, nodule characteristics, intra-procedure imaging modality, biopsy methods, diagnostic yield, sensitivity for malignancy, and safety data were analyzed.

## 3. Results

**Figure 1:**
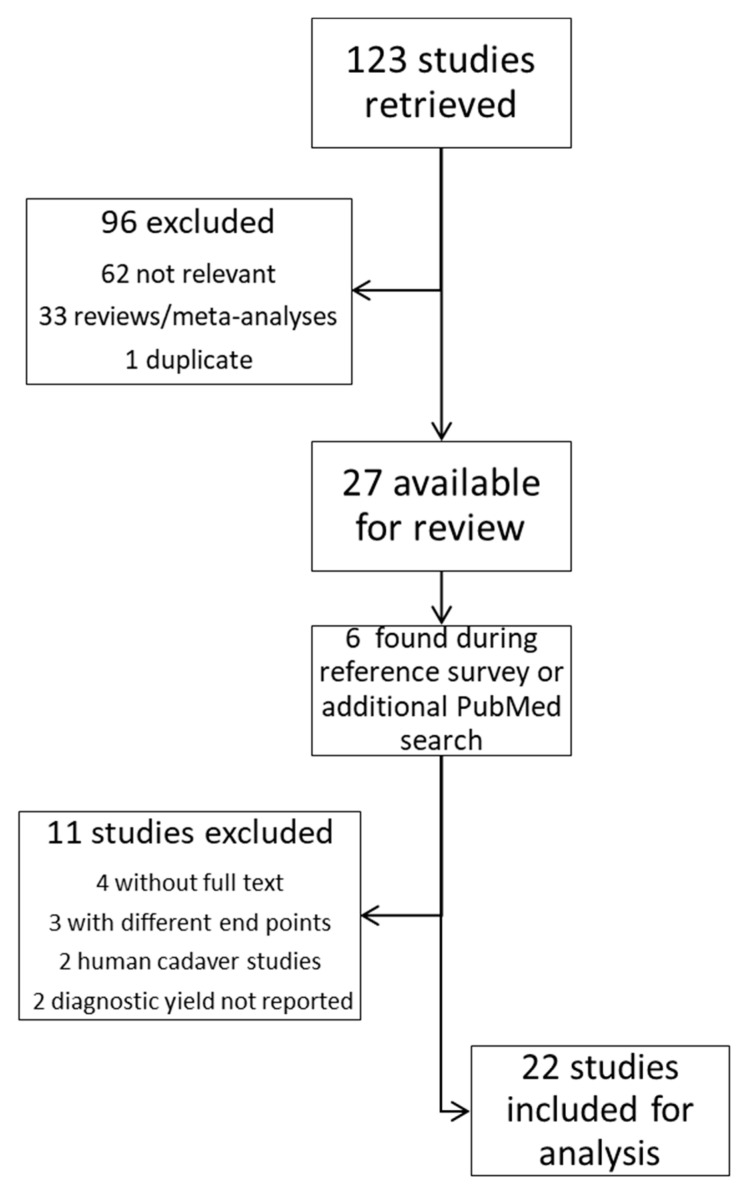
Review process of the included studies.

One hundred twenty-three articles were generated. Sixty-two were screened out as not relevant to the topic, thirty-three were review articles/meta-analyses and were excluded, and one duplicate entry was excluded. The remaining 27 entries were reviewed in full text. After the text review, 16 articles were included in the analysis, and 6 additional articles were found during a review of references and an additional search on PubMed. A total of 22 studies were available for the analyses (see Figure 1).

The smallest study was a retrospective trial that used the Ion^TM^ platform, which included eight patients [5]. The largest was a retrospective review published recently that analyzed the records of 407 patients [6]. Fifteen studies were retrospective, and sixteen studies were single-institution studies. Seventeen studies used the Ion^TM^ platform, including the largest study in the series [6].

### 3.1. Procedural Characteristics

Table 1 shows the most frequent imaging modalities and biopsy tools used in the reviewed studies. Radial endobronchial ultrasound (rEBUS) and 2D fluoroscopy were the most commonly used imaging modalities to confirm the catheter proximity to nodules. Few studies reported on the use of 3D “spin” fluoroscopy or cone beam CT, except when the authors were specifically investigating these modalities [7,8,9,10]. Transbronchial needle aspiration (TBNA) was the most common tool used, with a median of 96.95% and a range of 93.5–100%. The reported transbronchial forceps biopsy was more variable, with a median of 69.9% and ranging from 2.7 to 96%. The use of cryobiopsies in the literature was rare, but two studies reported improvements in the diagnostic yield with it [11,12].

### 3.2. Overall Diagnostic Yield and Sensitivity for Malignancy

Table 2 shows the included articles and their reported diagnostic yields, sensitivities, and specificities for malignancy, as well as positive and negative predictive values. The diagnostic yield was defined as the number of biopsies that resulted in a diagnosis of malignant or benign disease over the total number of biopsies performed [4]. The NAVIGATE trial followed nodules that were benign or indeterminate for 12 months after the biopsy to ensure the lesions were nonmalignant [4]. Hence, we indicate the follow-up time reported in each study in Table 2.

More recently, a consensus statement from American Thoracic Society/American College of Chest Physicians defined the diagnostic yield without the requirement of a pre-determined follow-up length [26]. Since 2022, there were 2 studies out of 17 reviewed here that appeared to use this definition [7,10]. One trial did define the diagnostic yield as pathology that matched the treatment recommendations from the institution’s multidisciplinary tumor board [7]. Overall, the diagnostic yield was variable and ranged from 69 to 93%, with a median of 86%. The sensitivity for malignancy across all studies ranged from 69% to 91.7%, with a median of 85%.

**Table 2:** List of the characteristics of the included articles. NA: not available or not reported.

Study	N	Design	Platform	Institution	Diagnostic Yield (%)	Sensitivity (%)	Specificity (%)	Positive Predictive Value (%)	Negative Predictive Value (%)	Length of Follow-Up (Months)	Study Country
Fielding et al., 2019 [22]	29	Prospective	Ion	Single	79.3	82.0	63.6	NA	NA	6	Australia
Chaddha et al., 2019 [21]	165	Retrospective	Monarch	Multi	69–77	NA	NA	NA	NA	6	United States
Kalchiem-Dekel 2021 [5]	8	Retrospective	Ion	Single	90	NA	NA	NA	NA	NA	United States
Benn et al., 2021 [8]	52	Prospective	Ion	Single	86	84–86	NA	NA	NA	5–16	United States
Chen et al., 2021 [20]	54	Prospective	Monarch	Multi	74.1	NA	NA	NA	NA	12	United States
Reisenauer et al., 2022 [7]	30	Prospective	Ion	Single	93.3	91.7	NA	NA	NA	NA	United States
Styrvoky et al., 2022 [17]	198	Retrospective	Ion	Single	89	87.3	98.7	99.2	81.3	14	United States
Oberg et al., 2022 [11]	112	Retrospective	Ion	Single	90.2	NA	NA	NA	NA	NA	United States
Kalchiem-Dekel et al., 2022 [16]	130	Prospective	Ion	Single	81.7	79.8	NA	NA	72.4	>12	United States
Lee-Mateus et al., 2023 [18]	113	Retrospective	Ion	Multi	87.6	82.1	100	100	71.4	12	United States
Vu et al., 2023 [19]	110	Retrospective	Ion	Single	NA	87	100	100	62	12	United States
Altaq et al., 2023 [15]	42	Retrospective	Ion	Single	88.1	86.5	100.	100.	50	12	United States
Agrawal et al., 2023 [13]	124	Retrospective	Monarch	Single	77	69	100	100	58	>12	United States
Khan et al., 2023 [14]	264	Retrospective	Monarch	Multi	77.3–79.9	74.2–79.3	NA	NA	NA	12	United States
Low et al., 2023 [24]	133	Retrospective	Ion	Single	77	NA	NA	NA	NA	NA	United States
Iwamoto et al., 2023 [23]	69	Retrospective	Monarch	Single	NA	90.5	100	100	86.7	12	United States
Brownlee et al., 2023 [6]	407	Retrospective	Ion	Single	87.9	NA	NA	100	91.6	NA	United States
Trimble et al., 2024 [27]	43	Retrospective	Ion	Single	86.1	NA	NA	NA	NA	24	United States
Abia-Trujillo et al., 2024 [9]	173	Retrospective	Ion	Multi	85.4	77.4	100	100	70.8	NA	United States
Bashour et al., 2024 [10]	67	Prospective	Ion	Single	86.6	98.9	100	NA	NA	12	United States
Abia-Trujillo et al., 2024 [12]	256	Retrospective	Ion	Single	70.4–91.	79.3–96	100			3	United States
Xie et al., 2024 [25]	90	Prospective	Ion	Multi	87.8	87.7	NA	NA	NA	6	China

**Figure 2:**
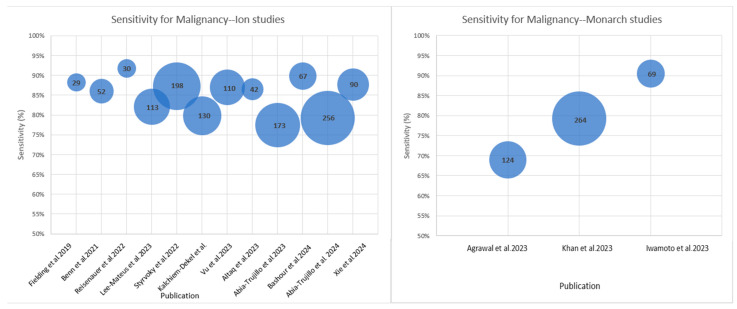
Reported sensitivity for malignancy according to platform [7,8,9,10,12,13,14,15,16,17,18,19,22,23,25].

As the Ion^TM^ and Monarch^TM^ platforms were the two most commonly used in the literature, we felt it prudent to analyze the outcomes stratified by platform. Figure 2 displays the sensitivity for the malignancy of studies according to which platform was used. Of the five studies that employed the Monarch^TM^ platform, two did not report a sensitivity for malignancy. The sensitivity for malignancy for the Ion^TM^ studies ranged from 79.3 to 91.7% [7,8,9,10,12,15,16,17,18,19,22,25], while the Monarch^TM^ studies ranged from 69 to 90.5% [13,14,23].

### 3.3. Factors Associated with Increasing Diagnostic Yield

Studies were grouped according to whether a characteristic was shown to correlate with increasing diagnostic yield (*p* < 0.05), whether there was a trend toward increasing yield (0.05 < *p* < 0.2), or whether there was no correlation (*p* > 0.2). Four studies demonstrated no correlation between the patient age, sex, or body mass index (BMI) and the diagnostic yield [13,14,15,19].

#### 3.3.1. Nodule Characteristics

Table 3 displays nodule characteristics associated with the diagnostic yield. Five studies demonstrated that the nodule size was associated with higher diagnostic yields [6,12,13,14,16]. One paper showed a trend between the nodule size and diagnostic yield [21], and five did not show an association between the nodule size and the diagnostic yield [9,15,19,20,25]. Nine studies did not show a definitive association between the nodule CT imaging appearance (solid, ground glass, or semi-solid) and the diagnostic yield [6,9,12,13,14,15,19,21,25]. The lobar location of nodules did not correlate with an increased diagnostic yield in five reports [6,9,13,14,21]. The report from Kalchiem-Dekel et al. did show that nodules located in the medial two thirds of the lung were associated with a greater yield after a univariate analysis, but this association became a trend only upon a multivariate analysis [16]. A nodule’s standardized uptake value (SUV) on positron emission tomography (PET) did not definitively correlate with an increased diagnostic yield [15,19].

The number and the size of the circle reflect the number of patients included in each study.

**Table 3:** Nodule characteristics associated with diagnostic yield.

Nodule Variable	Significant (*p* < 0.05)	Trend (0.05 < *p* < 0.20)	Not Significant (*p* > 0.2)
Nodule size	Abia-Trujillo et al., 2024 [12] * Brownlee et al., 2024 [6] Agrawal et al., 2023 [13] Khan et al., 2023 [14] Kalchiem-Dekel et al., 2022 [16] (univariate and multivariate analyses)	Chaddha et al., 2019 [21]	Abia-Trujillo et al., 2024 [9] Xie et al., 2024 [25] Altaq et al., 2023 [15] Vu et al., 2023 [19] Chen et al., 2021 [20]
Lesion appearance (solid, GG, semi-solid)		Vu et al., 2023 [19] **	Abia-Trujillo et al., 2024 [12] * Xie et al., 2024 [25] Abia-Trujillo et al., 2024 [9] Brownlee et al., 2023 [6] Agrawal et al., 2023 [13] Altaq et al., 2023 [15] Khan et al., 2023 [14] Chaddha et al., 2019 [21]
Location—periphery lung			Abia-Trujillo et al., 2024 [9] Brownlee et al., 2023 [6] Khan et al., 2023 [14] Chaddha et al., 2019 [21]
Location—medial two-thirds of lung	Kalchiem-Dekel et al., 2022 [16] (univariate analysis)	Agrawal et al., 2023 [13] Kalchiem-Dekel et al., 2022 [16] (multivariate analysis)	
Lobar location		Xie et al., 2024 [25]	Abia-Trujillo et al., 2024 [9] Brownlee et al., 2023 [6] Agrawal et al., 2023 [13] Khan et al., 2023 [14] Chaddha et al., 2019 [21]
SUV		Altaq et al., 2023 [15]	Vu et al., 2023 [19]

GG: ground glass. SUV: standardized uptake value. * Results pertain only to fine needle aspiration. ** *p* < 0.2 for needle biopsies, but >0.2 for forceps biopsies.

#### 3.3.2. Intraprocedural Findings

Table 4 displays the intraprocedural findings associated with the diagnostic yield. Obtaining any view on rEBUS was associated with a higher diagnostic yield in two papers [13,25]. Obtaining a concentric view on rEBUS during the procedure was shown to positively correlate with the diagnostic yield compared with obtaining an eccentric view in two studies [21,24]. Four other papers reported either a trend or no significant difference between the concentric and eccentric views [14,16,20,25]. The presence of a bronchus sign correlated with the diagnostic yield in five papers [6,9,13,21,24], there was a trend toward an increased yield in two papers [14,16], and there was no association with an increased diagnostic yield in four papers [12,15,20,25].

### 3.4. Adverse Events

**Figure 3:**
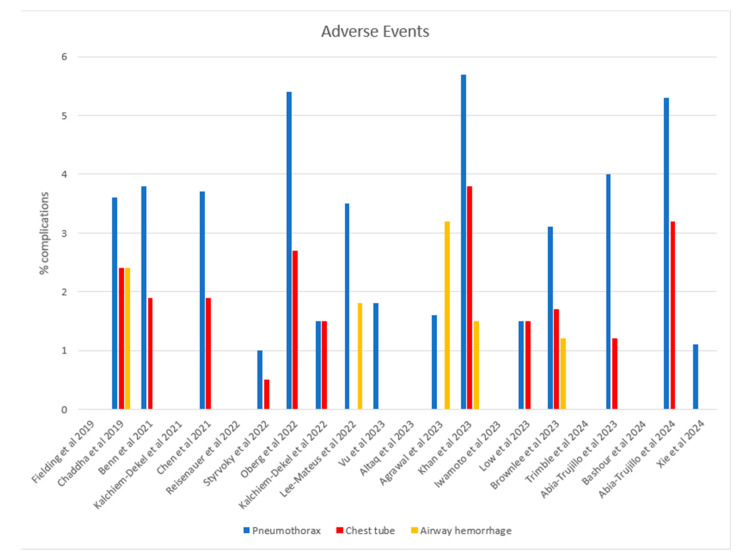
Adverse events related to robotic bronchoscopy [5,6,7,8,9,10,11,12,13,14,15,16,17,18,19,20,21,22,23,24,25,27].

The most common complication was a pneumothorax, which occurred between 1 and 5.7% of the time, with a median of 1.6%. An airway hemorrhage was the second most common adverse event, which occurred in 1.5–3.2% of cases. No deaths were reported (Figure 3).

## 4. Discussion

Flexible bronchoscopy has been instrumental in diagnosing pulmonary nodules for decades, but has been limited by the scope size, length, maneuverability, and the ability to easily pass tools through the bronchoscope [28]. Moreover, the diagnostic yield for flexible bronchoscopy sampling small (<2 cm) peripheral nodules is less than 50% [29]. Consequently, transthoracic biopsy approaches, with diagnostic yields of over 90%, have historically been widely utilized for sampling PPNs [2,30]. While the diagnostic yields have been higher, the pneumothorax rate has ranged between 18 and 25% [31].

The reviewed literature demonstrates that robotic platforms achieve a reasonably high diagnostic yield with fewer complications compared with TTNA. However, there are important caveats to this data. The article from Benn et al. noted a navigation success rate of 100% but a diagnostic yield of 86% [8]. This could be explained through CT–body divergence or the misclassification of true malignancies as alternative diagnoses, which was previously discussed [32]. Indeed, the definition of diagnostic yield was variable across the included studies. The NAVIGATE trial followed nonmalignant lesions for 12 months to ensure stability before finalizing their diagnoses as benign [4]. Among the studies reviewed, 12 had a follow-up time of 12 months or longer [8,10,13,14,15,16,17,18,19,20,23,27], while the remainder did not have a follow-up or did not specify a timeframe. This raises questions about the generalizability of the diagnostic yield data and points toward the necessity of a standardized definition of and timeframe for the follow-up of nonmalignant nodules [32,33]. With the advent of new guidelines for defining a diagnostic yield, it is possible that reported diagnostic yields may fluctuate in the literature going forward.

The reviewed studies provided mixed results on the relationship between the nodule size and diagnostic yield. Some of the discrepancies may have stemmed from arbitrary size distinctions. The studies from Khan et al. [14] and Agrawal et al. [13] reported increased diagnostic yields when the target nodule was at least 2 cm in diameter, while the study from Kalchiem-Dekel et al. in 2022 reported the same for nodules of at least 1.8 cm [16]. Conversely, the study by Chen et al. found that the nodule size did not predict the diagnostic yield and used a size cutoff of 3 cm. The largest study reviewed reported an increased diagnostic yield with every 1 mm increase in size [6]. Determining an absolute size cutoff that predicts higher diagnostic yields remains elusive.

The upper lobe location has been described as negatively affecting the diagnostic yield [4,34]. Operators often encounter tip-bend issues as they traverse more acute angles to reach the target nodule, which also makes catheter migration more likely after navigation. It is notable that in 17 articles in this review, the majority of the targeted nodules were in the upper lobes. Despite this, five studies found that the lobar location did not significantly impact the diagnostic yield [6,9,13,14,21]. Likewise, there is no definitive evidence that nodules within the medial two-thirds of the lung correlate with an increased diagnostic yield. The study by Kalchiem-Dekel in 2022 found a statistically significant relationship here, which then degraded into a trend only upon multivariate analysis [16].

Nine studies reviewed here did not find a significant difference between the nodule appearance on CT imaging and the diagnostic yield [6,9,12,13,14,15,19,21,25]. In contrast, prior work demonstrated decreasing diagnostic yields for TTNA, with increasing ground glass components within a nodule [35,36]. Earlier studies using the electromagnetic bronchoscopy platforms reported higher diagnostic yields when sampling nodules with a bronchus sign [37]. In this series, this relationship was less clear, as six studies did not find a clear association [12,14,15,16,20,25], while five did [6,9,13,21,24]. This may reflect bronchoscopists’ growing experience and comfort using the robotic platforms to biopsy PPNs.

As robotic bronchoscopy becomes more widespread, institutions will inevitably face choices as to which platform to purchase. Platform specifics and technical comparisons of the Ion^TM^ and Monarch^TM^ platforms were previously published [32,38]. To date, there has been no published trial that directly compared biopsy outcomes or adverse events between the Ion^TM^ and Monarch^TM^ consoles. This review provided some limited information to the prospective robotic platform purchaser. Three trials using Monarch^TM^ reported a sensitivities for malignancy of 69–90.5%. The trials that used the Ion^TM^ platform reported a sensitivities for malignancy of 79–91% (Figure 2). Although the figures were similar, this clearly represents an area for future studies.

There are important limitations of this review. There may exist a publication bias, where series that did not demonstrate a sufficiently high diagnostic yield or sensitivity for malignancy would perhaps not be published. Most of the studies were retrospective, including the largest study to date that involved robot-assisted platforms. Additionally, the majority of the reviewed studies relied on data from a single institution. We feel this is expected as the pulmonary community first had to demonstrate that robotic platforms were safe and effective through the reporting of smaller series. As the technology becomes more scrutinized and widespread, we anticipate more prospective, multi-institutional studies will be forthcoming. There is clearly heterogeneity in reporting the nodule size cutoffs, follow-up time for following benign or indeterminate lesions, and even the definition of the diagnostic yield. Rather than exclude studies for idiosyncratic definitions or cutoffs, we sought to describe the current literature and highlight practice differences to better inform the reader.

## 5. Conclusions

Robot-assisted bronchoscopy was demonstrated to be a safe method for sampling PPNs and achieved diagnostic yields that approached those of TTNAs. The nodule location may not affect the diagnostic yield, but the literature on this topic is mixed. Obtaining a concentric view on rEBUS, or at least an eccentric view, is likely associated with an increased diagnostic yield. Additional prospective studies, particularly comparing commercially available robotic platforms, would better inform practitioners and institutions as they adopt this new technology.

## Figures and Tables

**Table 1:** Imaging modalities and biopsy tools used.

**Imaging Modality Used (%)**	**Agrawal et al.** [13]	**Khan et al.** [14]	**Altaq et al.** [15]	**Kalchiem-Dekel et al., 2022** [16]	**Styrovsky et al.** [17]	**Lee-Mateus et al.** [18]	**Reisenauer et al.** [7]	**Vu et al.** [19]	**Chen et al.** [20]	**Benn et al.** [8]	**Chaddha et al.** [21]	**Fielding et al.** [22]
rEBUS	99.2	93.9	100	85.5	100	100	100	100	100	0 ^a^	100	96.6 ^b^
2D fluoroscopy	100	99.6	100	79.9		100	100	100		0	100	100
3D “spin” fluoroscopy				20.1			100 ^c^					
Cone beam CT		3.4			98.6					100		
**Biopsy Tools Used (%)**												
Needle aspiration	93.5	96.6		96.9		100		97		100	100	96.6
Forceps	94.4	70.8		32.1		2.7		68		76	96	69
Brush		17.1		2.5								75.9
BAL			100									86.2
Number of biopsies			10 ^d^									2.6 ^e^
**Imaging Modality Used (%)**	**Abia-Trujillo et al., 2024 ^f^ [9]**	**Brownlee et al. [6]**	**Iwamoto et al. [23]**	**Low et al. [24]**	**Oberg et al. ^g^ [11]**	**Bashour et al. ^h^ [10]**	**Abia-Trujillo et al., 2024 ^i^ [12]**	**Xie et al. [25]**				
rEBUS	99	57.4	0	100	100	0		100				
2D fluoroscopy	39.1	100		100	100	100		100				
3D “spin” fluoroscopy												
Cone beam CT	60.9		0			100						
**Biopsy Tools Used (%)**												
Needle aspiration	92.8			100			92.9					
forceps	41.7	94.7										
Brush	7.3					37						
BAL	56.8											
Cryobiopsy							100					

^a^ Did not use rEBUS or 2D fluoroscopy per the study protocol. ^b^ The rEBUS view was not reported for a single patient, so this was treated as if rEBUS had not occurred. ^c^ This was by study design to measure the tool for lesion divergence from the pre-op CT. ^d^ Median. ^e^ Mean. ^f^ Compared mobile cone beam CT with a standard C-arm. ^g^ Compared the use of cryobiopsy with traditional biopsy methods. ^h^ Compared the addition of cone beam CT with a robotic bronchoscopy without rEBUS use. ^i^ Compared cryobiopsy with fine needle aspiration.

**Table 4:** Procedural variables associated with diagnostic yield.

Procedure Variable	Significant (*p* < 0.05)	Trend (0.05 < *p* < 0.20)	Not Significant (*p* > 0.2)
rEBUS view: eccentric/concentric vs. no view	Xie et al., 2024 [25] Agrawal et al., 2023 [13]		
rEBUS view: concentric vs. eccentric	Low et al., 2023 [24] Chaddha et al., 2019 [21]	Kalchiem-Dekel et al., 2022 [16] (univariate analysis)	Xie et al., 2024 [25] Khan et al., 2023 [14] Chen et al., 2021 [20]
Bronchus sign present	Abia-Trujillo et al., 2024 [9] Low et al., 2023 [24] Brownlee et al., 2023 [6] Agrawal et al., 2023 [13] Chaddha et al., 2019 [21]	Khan et al., 2023 [14] Kalchiem-Dekel et al., 2022 [16] (univariate analysis)	Abia-Trujillo et al., 2024 [12] * Xie et al., 2024 [25] Altaq et al., 2023 [15] Chen et al., 2021 [20]

rEBUS: radial endobronchial ultrasound. * Results pertain only to fine needle aspiration.

## Data Availability

The data that support the findings of this study are available from the University of Oklahoma but restrictions apply to the availability of these data. Data are however available from the authors upon reasonable request and with the permission of the University of Oklahoma.

## Abbreviations

The following abbreviations are used in this manuscript:

PPN	Peripheral pulmonary nodule
LDCT	Low-dose chest computed tomography
TTNA	Transthoracic needle aspiration
RAB	Robot-assisted bronchoscopy
EMN	Electromagnetic
TBNA	Transbronchial needle aspiration
EBUS	Endobronchial ultrasound
SUV	Standardized uptake value
REBUS	Radial endobronchial ultrasound
